# Performance of copy number variants detection based on whole-genome sequencing by DNBSEQ platforms

**DOI:** 10.1186/s12859-020-03859-x

**Published:** 2020-11-11

**Authors:** Junhua Rao, Lihua Peng, Xinming Liang, Hui Jiang, Chunyu Geng, Xia Zhao, Xin Liu, Guangyi Fan, Fang Chen, Feng Mu

**Affiliations:** 1 MGI, BGI-Shenzhen, Shenzhen, 518083 China; 2 BGI-Shenzhen, Shenzhen, 518083 China; 3 BGI-Qingdao, BGI-Shenzhen, Qingdao, 266555 Shandong China; 4 MGI-Wuhan, BGI-Shenzhen, Wuhan, 430074 China; 5 China National GeneBank, BGI-Shenzhen, Shenzhen, 518120 China; 6 IGDB-BGI Joint Center for Omics, BGI-Shenzhen, Shenzhen, 518083 China; 7 State Key Laboratory of Agricultural Genomics, BGI-Shenzhen, Shenzhen, 518083 China

**Keywords:** Copy number variant (CNV), Whole-genome sequencing (WGS), DNBSEQ, Benchmark

## Abstract

**Background:**

DNBSEQ™ platforms are new massively parallel sequencing (MPS) platforms that use DNA nanoball technology. Use of data generated from DNBSEQ™ platforms to detect single nucleotide variants (SNVs) and small insertions and deletions (indels) has proven to be quite effective, while the feasibility of copy number variants (CNVs) detection is unclear.

**Results:**

Here, we first benchmarked different CNV detection tools based on Illumina whole-genome sequencing (WGS) data of NA12878 and then assessed these tools in CNV detection based on DNBSEQ™ sequencing data from the same sample. When the same tool was used, the CNVs detected based on DNBSEQ™ and Illumina data were similar in quantity, length and distribution, while great differences existed within results from different tools and even based on data from a single platform. We further estimated the CNV detection power based on available CNV benchmarks of NA12878 and found similar precision and sensitivity between the DNBSEQ™ and Illumina platforms. We also found higher precision of CNVs shorter than 1 kbp based on DNBSEQ™ platforms than those based on Illumina platforms by using Pindel, DELLY and LUMPY. We carefully compared these two available benchmarks and found a large proportion of specific CNVs between them. Thus, we constructed a more complete CNV benchmark of NA12878 containing 3512 CNV regions.

**Conclusions:**

We assessed and benchmarked CNV detections based on WGS with DNBSEQ™ platforms and provide guidelines for future studies.

## Background

Large structural variations in the human genome, especially copy number variants (CNVs), have been widely studied, and their important roles in human diseases, such as autism [1–4], schizophrenia [5], Parkinson’s disease [6], Hirschsprung disease [7] and cancer [8], have been clearly demonstrated. In addition, different technologies and methods have been used or developed to detect CNVs. Initially, fluorescence in situ hybridization (FISH), array-based comparative genomic hybridization (array CGH) [9, 10] and single nucleotide polymorphism (SNP) arrays were used. Then, with improvements in sequencing technologies, whole-genome sequencing (WGS) and whole-exome sequencing (WES) became more widely used in detecting CNVs without the limitations of specified target regions associated with hybridization or arrays.

Several tools for CNV detection were developed based on WES [11] or WGS [12, 13] data by Illumina platforms. Within these tools, there are five main strategies considered: (1) read pair (RP), (2) read depth (RD), (3) split read (SR), (4) de novo assembly (AS), and (5) combination of approaches (CA). Each strategy has its own advantages and limitations. RD-based tools can call accurate CNVs but are limited to detecting only the breakpoints of CNVs. SR-based tools can detect breakpoints at base-pair resolution but perform poorly in repetitive regions [12]. AS-based tools can detect CNVs without a known reference but require more computational resources [12]. Thus, different tools were designed for different samples and sequencing strategies. For example, BreakDancer [14] applies the RP strategy and is suitable for CNV detection in a single sample, while HYDRA [15], based on the CA strategy, is suited for multiple samples, and CNAnorm [16], based on the RD strategy, is designed for case–control studies.

DNBSEQ™ sequencing technology, developed by MGI Tech Co., Ltd. (MGI), was applied in different sequencing platforms, including BGISEQ-500, DNBSEQ-G400 and DNBSEQ-T7. Different from other sequencing technologies, DNBSEQ™ combines the technologies of DNA nanoballs (DNBs) with low amplification error rates, a high density patterned array and combinational Probe-Anchor Synthesis (cPAS) [17]. With these technologies, DNBSEQ™ sequencing platforms generate data with high sequencing accuracy, low duplication rates and reduced index hopping [18]. Previously, we explored the performances of single nucleotide variant (SNV) and small insertion and deletion (indel) detection on DNBSEQ™-based WGS data [19, 20], while the performances of CNV detection remained unexplored. Several benchmarking analyses of CNV detection on WGS data by Illumina platforms have been reported [6, 21]; thus, it is important to understand the performance of DNBSEQ™ with respect to CNV detection in comparison with Illumina platforms with various CNV tools, so users of DNBSEQ™ can choose the correct tools according to their needs. Here, we present the detection and performance evaluation of CNVs based on WGS data sequenced on DNBSEQ™ platforms.

## Results

### Detecting CNVs based on Illumina WGS data with different tools

**Fig. 1:**
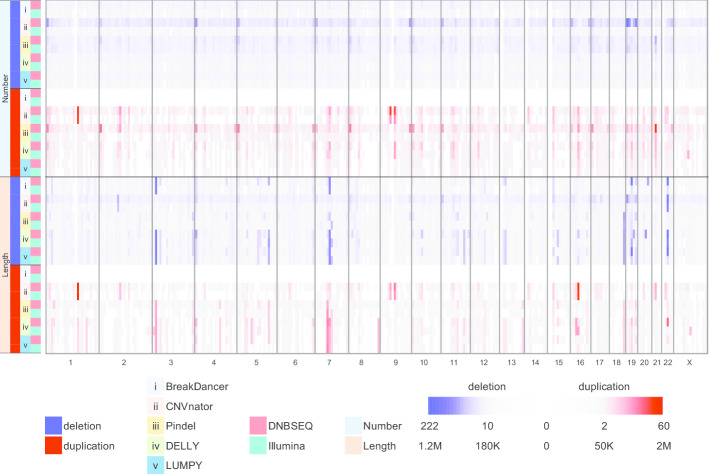
Heatmap of CNV results across the genome. The numbers and lengths of deletions and duplications detected on ten WGS datasets by five tools is profiled through 10 Mb bins across the whole genome. The colour bar on the left indicates the number and length panel, the deletion and duplication, five tools and two platforms. The 23 chromosomes are arranged linearly on the x-axis. (i) BreakDancer; (ii) CNVnator; (iii) Pindel; (iv) DELLY; (v) LUMPY

Using various CNV detection tools for Illumina WGS data, we selected five representative tools (BreakDancer [14], CNVnator [22], Pindel [23], DELLY [24] and LUMPY [25]) that are commonly used and were recently updated (see “Methods” for more details) for detecting CNVs based on a single WGS sample. We detected CNVs on two Illumina WGS datasets of NA12878, with an average depth of 30.61X (31.91X on HiSeq2500_PE150 and 29.30X on NovaSeq6000_PE150, Additional file 1: Table S1). We obtained 933 (968 and 897) CNVs on average using BreakDancer (see “Methods” for more details), 1945 on average (2660 and 1229) using CNVnator, 4888 on average (4045 and 5730) using Pindel, 1741 on average (1709 and 1773) using DELLY and 1365 on average (1380 and 1350) using LUMPY (Table 1, Fig. 1 and Additional file 1: Table S2). The consistency ratios between CNVs on Illumina platforms were 85.50% using BreakDancer, 55.77% using CNVnator, 53.90% using Pindel, 74.38% using DELLY and 83.49% using LUMPY (Additional file 2: Fig. S1). To compare and confirm features of CNVs detected by different tools, we first compared the numbers and lengths of the detected CNVs. We found that on average, 90.22% (8819/9775) of CNVs detected by Pindel were shorter than 1000 bp (Additional file 2: Fig. S2). This was consistent with previous findings that showed that Pindel is an effective tool to detect small CNVs [12]. Moreover, we found that on average, 99.20% (3858/3889) of CNVs were longer than 1000 bp using CNVnator (Additional file 2: Fig. S2). This indicated that CNVnator was more suitable for large CNV detection, consistent with a previous report [12]. Furthermore, using BreakDancer, DELLY and LUMPY, we obtained similar length distributions, which mostly fell into the range from 100 to 5000 bp (Additional file 2: Fig. S2).

**Table 1:** Statistics of CNV number and overall length of all 50 CNV sets

Dataset	Number					Overall length (Mb)				
	BreakDancer	CNVnator	Pindel	DELLY	LUMPY	BreakDancer	CNVnator	Pindel	DELLY	LUMPY
BGISEQ-500_PE100	2329	5778	4924	1904	2378	6.62	27.07	12.03	23.38	18.10
BGISEQ-500_PE150-1	1646	3617	4463	1624	1422	7.42	30.10	9.21	22.43	17.64
BGISEQ-500_PE150-2	2265	4241	4310	2209	2202	7.68	32.37	8.24	28.01	22.37
DNBSEQ-G400_PE100	2160	4354	4688	1745	1889	7.41	26.74	9.03	23.13	18.25
DNBSEQ-G400_PE150_PCR-1	1576	2234	4577	1902	1609	9.11	22.79	11.81	23.61	17.62
DNBSEQ-G400_PE150_PCR-2	1600	2209	4689	1900	1533	7.59	22.68	11.21	23.93	20.15
DNBSEQ-G400_PE150_PCRfree-1	1519	2890	4221	1705	1463	8.53	25.78	12.07	25.51	18.58
DNBSEQ-G400_PE150_PCRfree-2	1523	2860	4172	1713	1505	8.20	25.43	9.84	22.30	18.42
HiSeq2500_PE150	968	2660	4045	1709	1380	7.03	26.49	10.68	29.73	22.25
NovaSeq6000_PE150	897	1229	5730	1773	1350	6.32	22.54	9.64	28.52	22.02

We also analysed the regional distribution of detected CNVs across the genome. We found that CNVs detected by CNVnator were more enriched in exonic regions compared to those identified using the other four tools (average 41.43% vs. 28.98% and *P* = 0.007409 with the *t*-test) (Additional file 1: Table S3 and Additional file 2: Fig. S3), while the CNVs detected by Pindel were more enriched in intronic regions (average 32.63% vs. 23.33% with *P* = 3.406e−05 with the *t*-test). No notable bias was apparent in CNVs detected by BreakDancer, DELLY or LUMPY.

**Fig. 2:**
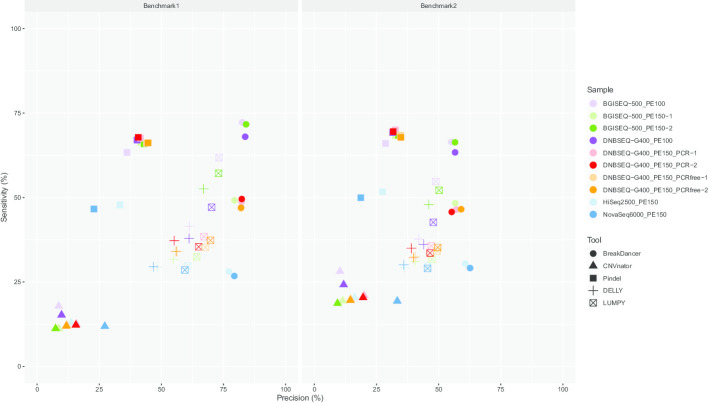
Evaluation of CNVs from 50 datasets on two benchmarks. Dot plot shows the sensitivity and precision of CNVs on ten datasets using five tools compared with those using Benchmark1 (left) and Benchmark2 (right). Ten datasets are marked with different colours, and five tools are marked with different point types. Benchmark1, data by Ryan et al. 2014; Benchmark2, data by Peter et al. 2015

Finally, we assessed the precision, sensitivity and F1-score of CNVs with these tools according to two benchmarks (data by Ryan et al. 2014, referred to as “Benchmark1” in this paper, and data by Peter et al. 2015, referred to as “Benchmark2”). We found that BreakDancer showed the highest precision (average 78.27% and 61.70% on Benchmark1 and Benchmark2, respectively) but low sensitivity (27.44% and 29.79%, respectively) and the highest F1-score (40.62% and 40.17%), while CNVnator had the lowest precision (20.47% and 24.78%, respectively), sensitivity (12.42% and 19.88%, respectively) and F1-score (14.93% and 21.28%, respectively) (Fig. 2). Meanwhile, Pindel showed low precision (28.11% and 23.08%, respectively) but the highest sensitivity (47.22% and 50.83%, respectively) and a high F1-score (35.00% and 31.54%, respectively), while both DELLY and LUMPY had moderate precision (47.57% and 36.10%, respectively, by DELLY and 60.03% and 45.60%, respectively, by LUMPY), sensitivity (29.45% and 29.66%, respectively, by DELLY and 29.13% and 29.35%, respectively, by LUMPY) and F1-score (36.38% and 32.56%, respectively, by DELLY and 39.22% and 35.71%, respectively, by LUMPY) (Fig. 2).

### Detecting CNVs based on DNBSEQ™ WGS data with different tools

We further detected CNVs on eight WGS datasets of NA12878 by DNBSEQ™ platforms, with an average depth of 31.43X (ranging from 29.51 to 37.44, Additional file 1: Table S1). We obtained 1827 (ranging from 1519 to 2329) CNVs on average using BreakDancer, 3523 (ranging from 2209 to 5778) on average using CNVnator, 4506 (ranging from 4172 to 4924) on average using Pindel, 1838 (ranging from 1624 to 209) on average using DELLY and 1750 (ranging from 1422 to 2378) on average using LUMPY (Table 1, Fig. 1 and Additional file 1: Table S2). We found high consistency ratios between these CNV sets on all DNBSEQ™ platforms except for CNVnator (56.54%), with an average of 75.96% CNVs consistent using BreakDancer, 73.19% using Pindel, 75.66% using DELLY and 76.99% using LUMPY (Additional file 2: Fig. S1). Moreover, in contrast to those based on Illumina platforms, we found similar length and regional distributions of CNVs based on DNBSEQ™ platforms (Additional file 1: Table S3 and Additional file 2: Figs. S2–3).

### Concordance of CNV between the DNBSEQ™ and Illumina platforms

Compared to Illumina platforms, we were able to detect more CNVs based on all DNBSEQ™ platforms except for Pindel (0.92-fold) using different tools (1.96-fold using BreakDancer, 1.81-fold using CNVnator, 1.06-fold using DELLY and 1.28-fold using LUMPY) (Table 1, Fig. 1). The average overall length of CNVs was similar: 1.17-fold using BreakDancer, 1.09-fold using CNVnator, 1.03-fold using Pindel, 0.83-fold using DELLY and 0.85-fold using LUMPY (Table 1). In brief, the consistency ratio of CNVs based on DNBSEQ™ platforms and Illumina platforms was, on average, 64.38% (ranging from 32.89 to 91.30%) using BreakDancer, 50.24% (ranging from 19.52 to 75.10%) using CNVnator, 57.66% (ranging from 43.61 to 67.99%) using Pindel, 68.85% (ranging from 54.69 to 77.71%) using DELLY and 72.32% (ranging from 46.05 to 82.96%) using LUMPY (Additional file 2: Fig. S1).

Furthermore, we annotated the regional distribution of the detected CNVs. We found similar distribution of these identified CNVs, with an average of 37.28% CNVs based on DNBSEQ™ platforms and 36.25% CNVs based on Illumina platforms in intergenic regions, 28.32% and 31.47%, respectively, in exonic regions, 27.49% and 25.19%, respectively, in intronic regions, 3.53% and 3.56%, respectively, in gene downstream regions, 3.38% and 3.53%, respectively, in gene upstream regions, 5.01% and 4.53%, respectively, in CpG island (CGI) shores, and 2.85% and 2.05%, respectively, in CGIs (Additional file 1: Table S3 and Additional file 2: Fig. S3).

### High precision of specific CNVs from DNBSEQ™ platforms

We evaluated the precision, sensitivity and F1-score of CNVs based on DNBSEQ™ platforms using both Benchmark1 and Benchmark2. The average precision, sensitivity and F1-score were 82.33%, 56.66%, and 66.60%, respectively, on Benchmark1 and 56.95%, 53.72%, and 54.84%, respectively, on Benchmark2 using BreakDancer, 11.24%, 13.07%, and 11.77% and 13.94%, 21.41%, and 16.46%, respectively, using CNVnator, 41.54%, 66.28%, and 51.04% and 32.56%, 68.48%, and 44.11%, respectively, using Pindel, 58.36%, 38.27%, and 46.13% and 41.37%, 35.95%, and 38.39%, respectively, using DELLY, and 68.81%, 43.12%, and 52.56% and 48.35%, 39.98%, and 43.37%, respectively, using LUMPY (Fig. 2). These results showed similar performance of CNV detection with the DNBSEQ™ and Illumina platforms across the genome.

**Fig. 3:**
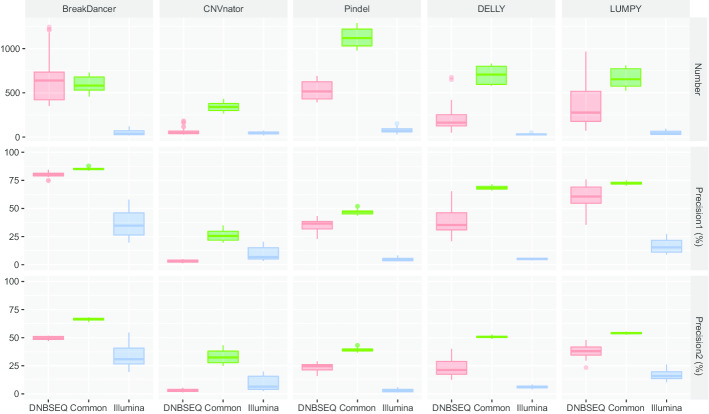
Comparison of CNVs by platforms. Box plot shows the number (upper), precision based on Benchmark1 (median) and precision based on Benchmark2 (lower) of common and specific CNVs between platforms by different tools (column). Precision1, precision based on Benchmark1; Precision2, precision based on Benchmark2; DNBSEQ, specific CNVs on DNBSEQ™ platforms; Illumina, specific CNVs on Illumina platforms; Common, common CNVs between the DNBSEQ™ and Illumina platforms

To further investigate and compare the accuracy of CNVs detected by DNBSEQ™ and Illumina platforms, we calculated the precision of common and specific CNVs between any two CNV sets. We found significantly higher precision of common CNVs than of specific CNVs by using five different tools (*P* < 2.2e−16 with the *t*-test, Fig. 3 and Additional file 2: Figs. S4–5). In the comparison between CNVs detected by DNBSEQ™ and Illumina platforms, we obtained similarly high consistency ratios of CNVs using Pindel (on average 59.36% and 55.95% consistent CNVs on DNBSEQ™ and Illumina platforms, respectively), DELLY (67.70% and 70.00%, respectively) and LUMPY (64.93% and 79.70%, respectively) but not BreakDancer (44.11% and 84.65%, respectively) or CNVnator (38.67% and 61.82%, respectively) (Fig. 3 and Additional file 2: Fig. S1). We also found higher precision of specific CNVs on DNBSEQ™ platforms than on Illumina platforms by using BreakDancer (average 64.23% of DNBSEQ™ vs. 31.78% of Illumina), Pindel (27.90% vs. 3.60%), DELLY (29.78% vs. 5.54%) and LUMPY (45.84% vs. 15.85%) but not CNVnator (3.25% vs. 6.82%) (Fig. 3).

**Fig. 4:**
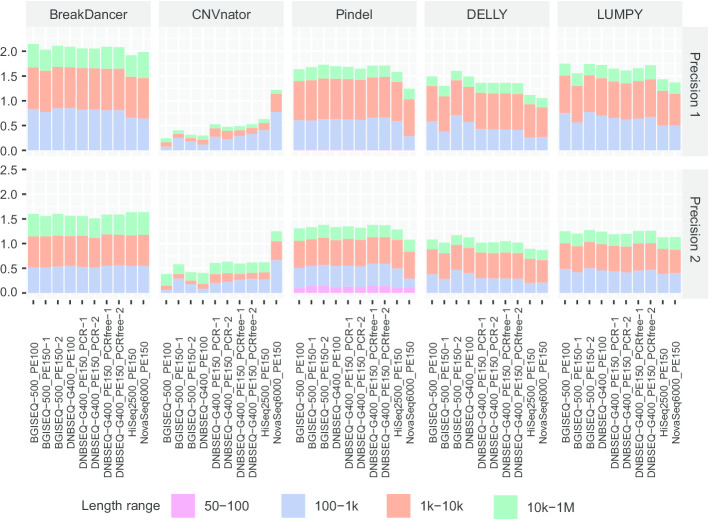
Precision of CNVs with different length ranges. Histogram shows the precision of CNVs detected by different tools (column) based on different benchmarks (row). Precision for CNVs is shown with different colour bars according to length ranges. Precision1, precision based on Benchmark1; Precision2, precision based on Benchmark2

Moreover, we divided CNVs into four groups according to their length (50–100 bp, 100 bp-1 kbp, 1–10 kbp and 10 kbp-1 Mbp) and evaluated the precision of CNVs in these groups. We found higher precision of CNVs in the 100 bp-1 kbp length group from DNBSEQ™ platforms than those from Illumina platforms by Pindel (61.15% on average from DNBSEQ™ platforms vs. 28.05% on average from Illumina platforms using Benchmark1, 42.26% vs. 19.09% using Benchmark2, respectively), DELLY (42.92% vs. 26.75% and 29.68% vs. 20.92%, respectively) and LUMPY (65.85% vs. 50.04% and 45.83% vs. 39.35%, respectively) but not BreakDancer (80.97% vs. 64.47% and 52.13% vs. 54.55%, respectively) or CNVnator (14.86% vs. 40.91% and 13.28% vs. 27.27%, respectively) (Fig. 4). These results showed that CNVs with small lengths were more accurately detected from WGS data sequenced on DNBSEQ™ platforms.

### Constructing a complete CNV benchmark of NA12878

As described above, we found a versatile consistency ratio in pairwise comparisons between any two CNV sets (Additional file 2: Fig. S1). However, significant differences in precision (*P* = 3.57e−12 with the *t*-test, Additional file 2: Fig. S6) were observed between the two available CNV benchmarks. To construct a more complete CNV benchmark of NA12878, we constructed a predicted CNV benchmark from all 50 CNV sets by incorporating any CNV regions that were detected by at least two tools and two platforms. Then, we integrated the unions of the predicted CNV set and two available benchmarks (see “Methods” for more details). Ultimately, we produced a novel complete CNV benchmark of NA12878, named “Benchmark3”, with 3512 CNVs, including 3168 deletions and 344 duplications (Additional file 1: Table S4). We found that the length of CNVs in Benchmark3 ranged from 50 to 557,758 bp. Of these, 70.62% (2480/3512) were larger than 1000 bp (Additional file 2: Fig. S7a). Concerning the components of Benchmark3, 70.44% (2474/3512) of CNVs were derived from only two available benchmarks, and 7.60% (267/3512) were only predicted by ten WGS datasets in our study (Additional file 2: Fig. S7b). We used Benchmark3 to evaluate the CNVs of DNBSEQ™ platforms and Illumina platforms and found that the precision, sensitivity and F1-score obtained by using three benchmarks were consistent among different platforms (Additional file 2: Fig. S7c).

## Discussion

CNVs are important genome variants and have been reported to be important in causing diseases such as cancer. Because of their importance, sequencing data-based CNV detection can be widely applied with the development of sequencing technologies. Many tools based on WGS data were developed for CNV detection, and these tools were estimated with in-house simulated data or nonuniform real data, such as trios used in CNVnator [22] and NA18507 used in Pindel [23].

In this study, we explored the performances of different germline CNV detection methods based on different WGS data of NA12878. Our results showed that the consistency ratios of CNVs detected by the same tool (on average 68.45%) were higher than those detected by different tools (39.00%), consistent with previous research [12]. Furthermore, we found that the consistency ratios of CNVs of the same platform detected by BreakDancer (on average 76.29%), Pindel (72.52%), DELLY (75.61%) and LUMPY (77.21%) were higher than that detected by CNVnator (56.52%, Additional file 2: Fig. S1). Moreover, we found that the consistency rates of CNV results among different platforms detected by BreakDancer and CNVnator were quite different. Interestingly, we found that the CNVs detected by DELLY and LUMPY were highly consistent, either between tools (on average 72.20%) or within tools (74.34%), or between platforms (70.58%) or within platforms (76.41%), which shows the advantage of the unique features of multiple CA-based strategy tools [12]. These results suggest that DELLY and LUMPY might be good choices for germline CNV detection based on DNBSEQ™ platforms.

Furthermore, we introduced two CNV benchmarks of NA12878 to determine the precision of CNVs. The precision of consistent CNVs among different platforms was, on average, 75.91% using BreakDancer, 28.51% using CNVnator, 43.41% using Pindel, 59.43% using DELLY and 54.80% using LUMPY (Fig. 3). In addition, we found that the precision of specific CNVs with DNBSEQ™ platforms was higher than that with Illumina platforms using BreakDancer (average 64.23% of DNBSEQ™ vs. 31.78% of Illumina), Pindel (27.90% vs. 3.60%, respectively), DELLY (29.78% vs. 5.54%, respectively) and LUMPY (45.84% vs. 15.85%, respectively, Fig. 3). This result might be due to the reduction of amplification bias by DNBSEQ™ sequencing technology [18], and we will further verify this result in a follow-up study.

## Conclusion

Many comparative analyses of CNV detection tools based on arrays [26], WES [11] and WGS [12, 13, 27], have been published. Most WES and WGS data analyses were based on datasets sequenced on Illumina platforms. Until now, there was no comprehensive analysis of CNV detection based on datasets sequenced on DNBSEQ™ platforms. This study represents the first systematic investigation and characterization of CNV detection using WGS data based on DNBSEQ™ platforms with five representative tools. We found that the quantity, length, distribution, sensitivity and precision of CNVs across the genome detected on DNBSEQ™ datasets was comparable to those detected on Illumina datasets. We also found that DNBSEQ™ platforms provided a more accurate overview of small CNVs than Illumina platforms. We constructed a relatively complete CNV benchmark by integrating the union of CNV sets from different datasets detected by different tools and two public benchmarks of NA12878. In summary, our study provides a comprehensive guide for CNV researchers using DNBSEQ™ platforms with benchmarks and performance measures.

## Methods

### Published WGS data

All fastq data of NA12878 were downloaded from the following websites: GigaScience DataBase (GigaDB), The *National Center for Biotechnology Information* (NCBI) and the China National GeneBank Sequence Archive (CNSA). All data were downsampled to approximately 30 × and aligned to the human reference genome hg19 following our previous WGS approach [19]. Ten WGS datasets with an average coverage of 31.27x (ranging from 29.3 × to 37.44x), a high mapping rate (average 99.46%, ranging from 99.06 to 99.60%) and high genome coverage (average 99.02%, ranging from 98.97 to 99.12%) provide good foundations for CNV detection (Additional file 1: Table S1).

### Tools and parameters

There are approximately 44 published CNV detection tools based on WGS data distributed into five strategies (Additional file 1: Table S5). We carefully selected five representative tools while mainly considering four factors: single sample pattern, widely used, continuous updating and strategy. The selected tools were BreakDancer (RP strategy, ver. 1.4.5) [14], CNVnator (RD strategy, ver. 0.3.3) [22], Pindel (SR strategy, ver. 0.2.5b9) [23], DELLY (ver. 0.7.8) [24] and LUMPY (ver. 0.2.13) [25], which was built on the CA strategy. All tools were processed with default parameters, except the optimal bin size in CNVnator was chosen according to the authors’ recommendations such that the ratio of the average read-depth signal to its standard deviation was between 4 and 5.

### CNV benchmarks of NA12878

For the reference CNV benchmark, two available benchmarks of NA12878 were introduced for evaluating CNVs: 2819 CNVs by Ryan et al. 2014 (Benchmark1) [25] and 2171 CNVs by Peter et al. 2015 (Benchmark2) [28]. We carefully compared these two CNV benchmarks and found a large proportion of specific CNVs in both Benchmark1 and Benchmark2. When we defined a CNV in one benchmark as specific if it overlapped with any CNV in the other benchmark by < 90% reciprocally in size, we found that 56.05% of CNVs in Benchmark1 and 32.43% of CNVs in Benchmark2 were specific. If we set 50.00% as the threshold of reciprocal overlap according to size, 47.82% and 30.49% of CNVs in Benchmark1 and Benchmark2, respectively, were specific (Additional file 2: Fig. S8).

### CNV detection and filtration

The CNV set was detected on different datasets by different tools. First, to reduce the number of false positives, non-CNVs were filtered from the initial outputs.

The outputs from BreakDancer were filtered according to the following criteria: (1) SV type was not ‘DEL’, (2) confidence score < 90, (3) supporting read pairs < 3, (4) not autosomal or chrX, and (5) overlapping a gap in the reference genome.

The outputs from CNVnator were filtered according to the following criteria: (1) q0 ≥ 0.5 or q0 < 0, (2) e-val1 ≥ 0.05, (3) not autosomal or chrX, and (4) overlapping a gap in the reference genome.

The outputs from Pindel were filtered according to the following criteria: SVTYPE was not ‘DEL’ or ‘DUP:TANDEM’ and supporting read pairs < 3. Additionally, CNVs were filtered according to the following criteria: (1) not autosomal or chrX and (2) overlapping a gap in the reference genome.

The outputs from DELLY were filtered according to the following criteria: (1) SVTYPE was not ‘DEL’ or ‘DUP’, (2) FILTER was ‘LowQual’, and (3) supporting read pairs < 3.

The outputs from LUMPY were filtered according to the following criteria: (1) SVTYPE was not ‘DEL’ or ‘DUP’ and (2) PE < 3. Additionally, CNVs were filtered according to the following criteria: (1) not autosomal or chrX and (2) overlapping a gap in the reference genome.

Within each obtained CNV set, we further filtered the CNVs that overlapped with other CNVs in the same CNV set, as these scenarios probably indicated complex structure variations. We required at least 1 bp overlap among CNVs to define them as overlapping, and the remainder were defined as non-overlapping. We found that on average, 9.82% (ranging from 3.75 to 20.36%) of the CNVs detected by BreakDancer for ten datasets overlapped, which is notably less than that detected by Pindel (39.43%, ranging from 30.97 to 49.96%), DELLY (19.87%, ranging from 16.55 to 26.81%) and LUMPY (30.23%, ranging from 23.73 to 43.67%) (Additional file 2: Fig. S9a). Overlapping CNVs were not found in any of the ten datasets with CNVnator (Additional file 2: Fig. S9a). Next, we assessed the precision of overlapping CNVs and non-overlapping CNVs on two benchmarks. For Benchmark1, the precision of non-overlapping CNVs was significantly higher than that of overlapping CNVs (Additional file 2: Fig. S9b, average precision 51.17% of non-overlapping CNVs vs. average precision 12.83% of overlapping CNVs, *P* = 5.64e−13 with the *t*-test). Similarly, for Benchmark2, we also found that the precision of non-overlapping CNVs was significantly higher than that of overlapping CNVs (Additional file 2: Fig. S9c, average 39.23% vs. average 9.08%, *P* = 1.11e−16 with the *t*-test). These results clearly show the negative effect of overlapping CNVs, prompting us to remove the overlapping CNVs in the subsequent evaluation analysis.

### Comparison between CNV sets

Any two CNV sets were compared for common (or consistent) and specific CNVs and marked according to their platforms. For example, a comparison between BGISEQ-500_PE100 and DNBSEQ-G400_PE100 was marked as “DNBSEQ_vs_DNBSEQ”, and a comparison between BGISEQ-500_PE100 and HiSeq2500_PE150 was marked as “DNBSEQ_vs_Illumina”. A CNV in one CNV set was considered common if either it overlapped with a single CNV in another CNV set by ≥ 50% reciprocally in size or there existed a set of CNVs in another CNV set such that each CNV in another CNV set had ≥ 50% size overlap with the CNV and ≥ 50% of the CNV overlapped with this set of CNVs in another CNV set. The remainder were considered specific. All three parts (common, specific in one CNV set and specific CNVs in another CNV set) were evaluated with benchmarks.

### Evaluation of CNVs

The evaluation of CNVs based on each benchmark was performed with in-house scripts. CNVs were evaluated based on deletions and duplications separately. A CNV was considered valid if either it overlapped with a single CNV in a benchmark by ≥ 50% reciprocally in size or there existed a set of CNVs in a benchmark such that each CNV in the benchmark had ≥ 50% size overlap with the CNV and ≥ 50% of the CNV overlapped with this set of CNVs in the benchmark.

The precision, sensitivity and F1-score were calculated in each CNV set using the following equations:

$$\begin{aligned} Sensitivity & = TP/\left( {TP + FN} \right) \\ Precision & = TP/\left( {TP + FP} \right) \\ F1{ - }score & = 2* \frac{Precision*Sensitivity}{{Precision + Sensitivity}} \\ \end{aligned}$$

where $TP$ is the true positive of deletions and duplications, $FP$ is the false positive of deletions and duplications and $FN$ is the false negative of deletions and duplications.

### Construction of the complete CNV benchmark

All CNV regions were classified as deletions or duplications. For deletions or duplications, a fragment collection was built according to the breakpoints of CNV regions from all 50 CNV sets. Then, all fragments were classified according to the sequencing platform and the CNV detection tool. One fragment was classified as BGISEQ-500 or DNBSEQ-G400 by a tool if the fragment was detected in all datasets from the same platform (three datasets of BGISEQ-500 and five datasets of DNBSEQ-G400) by the same tool. A fragment was marked as a potential benchmark if it was supported by at least two platforms and at least two tools. A predicted benchmark was built by merging all potential benchmark fragments. Later, a novel CNV benchmark was built by integrating all unions of CNVs from the predicted benchmark, Benchmark1 and Benchmark2.

### Annotation of CNVs

Genomic regions and CGIs were downloaded from the UCSC Genome Browser (https://hgdownload.soe.ucsc.edu/goldenPath/hg19/database/). The 2000 bp flanking region of the CGI in each direction was defined as the CGI-shore. The region located − 2000 bp to 0 bp of the transcription start site (TSS) was defined as upstream, and the region located 0 bp to + 2000 bp of the transcription end site (TES) was defined as downstream. CNVs were located in any of seven regions (upstream, exonic, intronic, intergenic, downstream, CGI and CGI-shore) if the midpoint of the CNV region was in any region.

## Supplementary information

**Additional file 1. Table S1**: Whole-genome sequencing data of NA12878 based on four sequencing platforms. **Table S2**: Number and length statistics of all 50 CNV sets. T**able S3**: Regional distribution of all 50 CNV sets across the genome. **Table S4**: List of the complete CNV benchmark of NA12878. **Table S5**: CNV detection tools using whole-genome sequencing data.

**Additional file 2. Figure S1**: Consistency ratios of pairwise comparisons between all 50 CNV sets. Heatmap shows the consistency ratio distribution between any two CNV sets or benchmarks. **Figure S2**: Summary of the density distribution of CNV length for ten datasets using five tools. Each inner chart represents the CNV results of ten datasets detected by each tool. In each inner chart, the line plot shows the density (y-axis) of the CNV count at a certain CNV length (x-axis), and the two black vertical lines indicate the Alu elements (left) and the LINE1 elements (right). **Figure S3**: Annotation of CNVs across the genome. Histogram shows the number (upper) and proportion (lower) of CNVs occurring in different regions across the genome. CpG island: CGI. CpG island-shore: CGI-shore. **Figure S4**: Comparison of CNVs by data on DNBSEQTM platforms. Box plot shows the number (upper), precision based on Benchmark1 (median) and precision based on Benchmark2 (lower) of common and specific CNVs between platforms by different tools (column). Precision1, precision based on Benchmark1; Precision2, precision based on Benchmark2. **Figure S5**: Comparison of CNVs by data on Illumina platforms. Box plot shows the number (upper), precision based on Benchmark1 (median) and precision based on Benchmark2 (lower) of common and specific CNVs between platforms by different tools (column). Precision1, precision based on Benchmark1; Precision2, precision based on Benchmark2. **Figure S6**: Comparison of the precision and sensitivity between two benchmarks on all 50 CNV sets. (A) Box plot shows the difference in precision between Benchmark1 (left) and Benchmark2 (right). (B) Box plot shows the difference in sensitivity between two benchmarks. Boxplot represents the rate of all 50 CNV sets, and dashed lines were drawn to connect the results based on the same dataset. **, P < 0.01; measured by the t-test. Benchmark1, data by Ryan et al. 2014; Benchmark2, data by Peter et al. 2015. **Figure S7**: Novel, complete CNV benchmark of NA12878. (a) Histogram shows the number of CNVs with different lengths in the complete NA12878 CNV benchmark. (b) Pie shows the components of the complete NA12878 CNV benchmark. Labels without a plus sign, such as “Benchmark1”, “Benchmark2” and “Predicted”, represent a unique source of the CNV benchmark. Labels with a plus sign indicate that the CNV benchmark was provided by at least two sources (“Benchmark 1+Predicted” indicates that the CNV benchmark was provided by both Benchmark1 and Predicted benchmark). (c) Bar plot shows the precision, sensitivity and F1-score of DNBSEQTM platforms and Illumina platforms. Black lines show the standard error. Benchmark1, data by Ryan et al. 2014; Benchmark2, data by Peter et al. 2015; Predicted, a predicted CNV benchmark from CNVs in this study. **Figure S8**: Comparison of two CNV benchmarks. Venn diagram shows the number and ratio of specific CNVs in each benchmark with a 90.00% threshold (A) or a 50.00% threshold (B). Benchmark1, data by Ryan et al. 2014; Benchmark2, data by Peter et al. 2015. **Figure S9**: Summary of the distribution and precision of overlapping and non-overlapping CNVs. (a) Histogram shows the number (upper) and proportion (lower) of overlapping and non-overlapping CNVs (n=50) from ten datasets using five tools. (b, c) The comparison of precision between overlapping and non-overlapping CNVs with Benchmark1 (b) and Benchmark2 (c) is displayed below. Benchmark1, data by Ryan et al. 2014; Benchmark2, data by Peter et al. 2015.

## Data Availability

All data analysed during this study are included in this published article and its supplementary files. The sequence accession numbers for WGS data are listed in Additional file 1: Table S1.

## Abbreviations

MPS: Massively parallel sequencing
SNV: Single nucleotide variant
Indel: Insertion and deletion
CNV: Copy number variant
WGS: Whole-genome sequencing
WES: Whole-exome sequencing
FISH: Fluorescence in situ hybridization
array CGH: Array-based comparative genomic hybridization
SNP: Single nucleotide polymorphism
RP: Read pair
RD: Read depth
SR: Split read
AS: De novo assembly
CA: Combination of approaches
MGI: MGI Tech Co., Ltd.
DNB: DNA nanoball
cPAS: Combinational probe-anchor synthesis
CGI: CpG Island
