# Supplementary material for: Performance of copy number variants detection based on whole-genome sequencing by DNBSEQ platforms
Source: BMC Bioinformatics. 2020 Nov 11;21:518. doi: 10.1186/s12859-020-03859-x (PMC7659224; doi:10.1186/s12859-020-03859-x)

**Table of contents in Additional file 2**

**Figure S1**. Consistency ratios of pairwise comparison between all 50 CNV sets.

**Figure S2**. Summary of the density distribution of CNV length for ten datasets using five tools.

**Figure S3**. Annotation of CNVs across the genome.

**Figure S4.** Comparison of CNVs by data on DNBSEQ^TM^ platforms.

**Figure S5.** Comparison of CNVs by data on Illumina platforms.

**Figure S6.** Comparison of the precision and sensitivity between two benchmarks on all 50 CNV sets.

**Figure S7.** Novel, complete CNV benchmark of NA12878.

**Figure S8.** Comparison of two CNV benchmarks.

**Figure S9.** Summary of the distribution and precision of overlapping and non-overlapping CNVs.

**Figure S1.** Consistency ratios of pairwise comparisons between all 50 CNV sets. Heatmap shows the consistency ratio distribution between any two CNV sets or benchmarks.


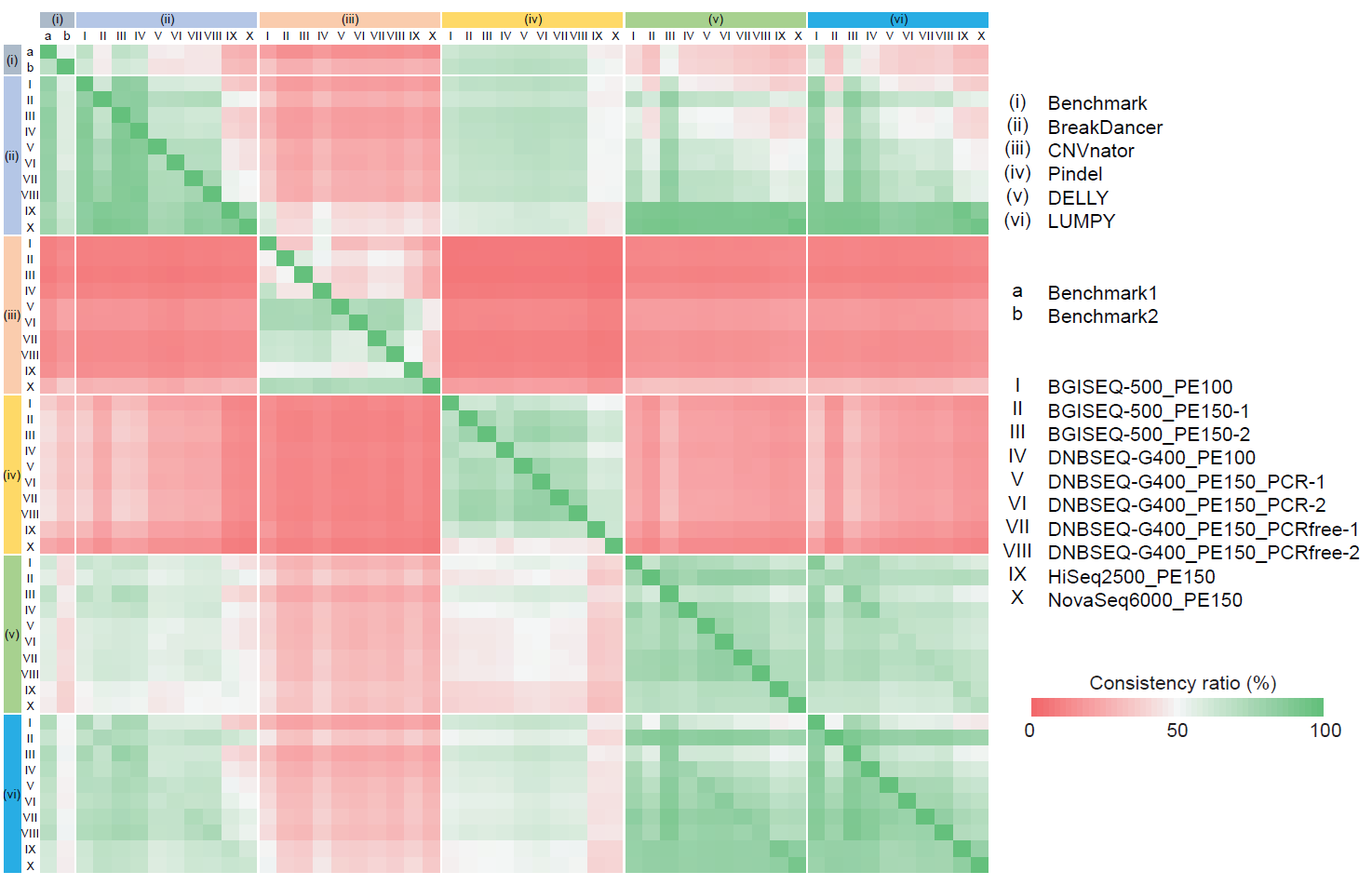


**Figure S2.** Summary of the density distribution of CNV length for ten datasets using five tools. Each inner chart represents the CNV results of ten datasets detected by each tool. In each inner chart, the line plot shows the density (y-axis) of the CNV count at a certain CNV length (x-axis), and the two black vertical lines indicate the Alu elements (left) and the LINE1 elements (right).


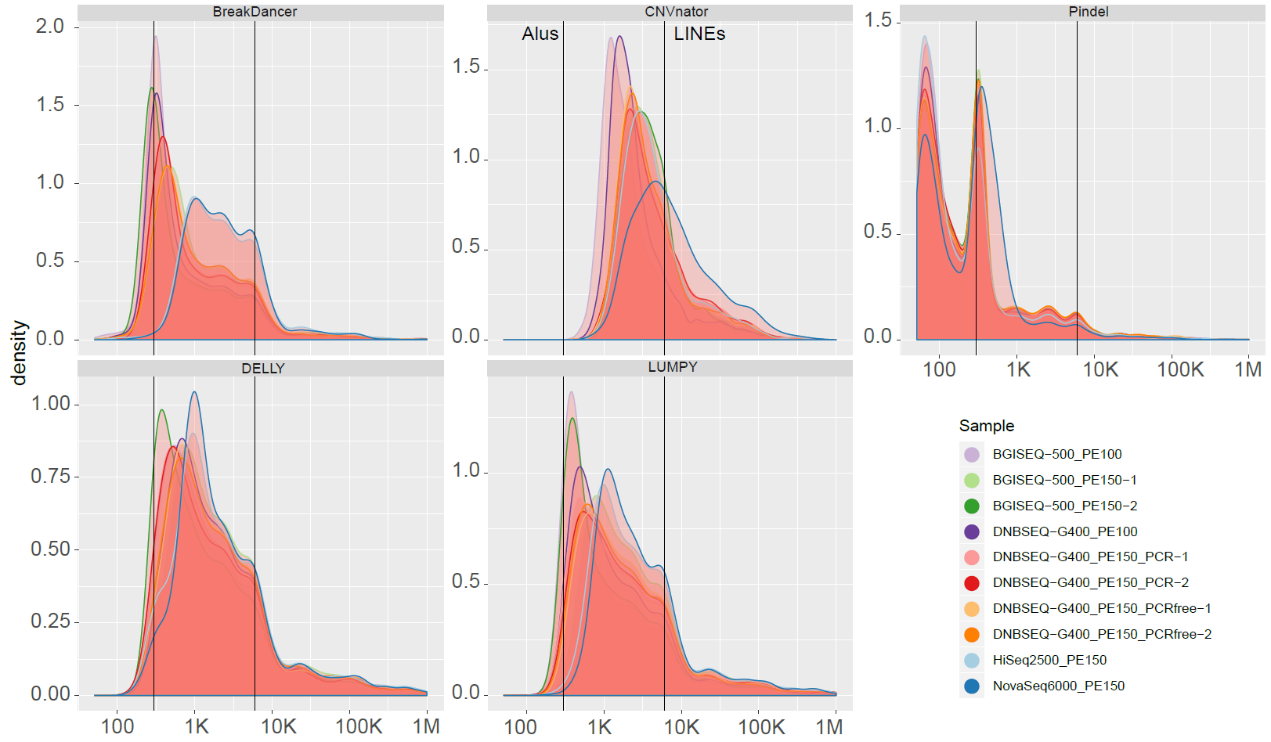


**Figure S3.** Annotation of CNVs across the genome. Histogram shows the number (upper) and proportion (lower) of CNVs occurring in different regions across the genome. CpG island: CGI. CpG island-shore: CGI-shore.


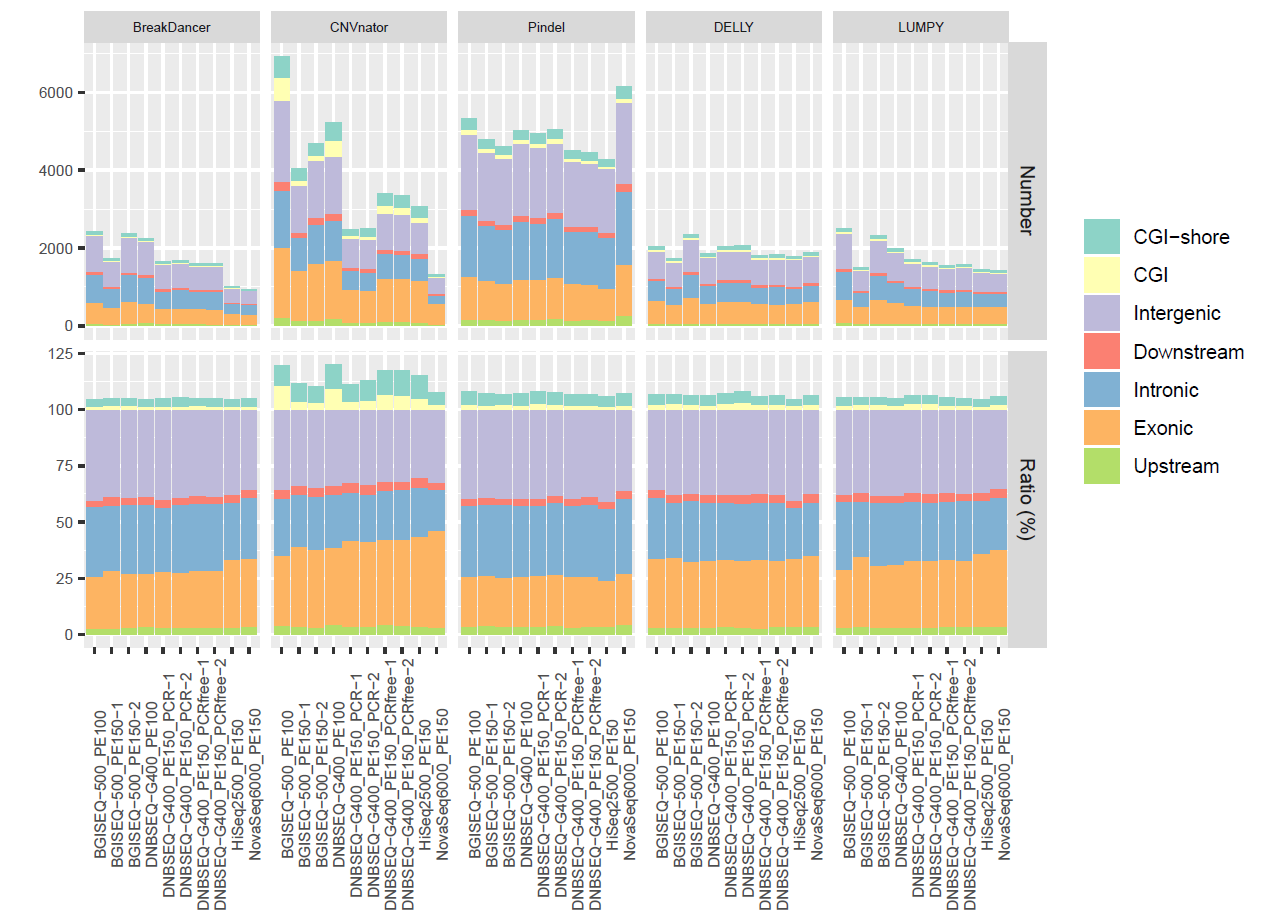


**Figure S4**. Comparison of CNVs by data on DNBSEQ^TM^ platforms. Box plot shows the number (upper), precision based on Benchmark1 (median) and precision based on Benchmark2 (lower) of common and specific CNVs between platforms by different tools (column). Precision1, precision based on Benchmark1; Precision2, precision based on Benchmark2.


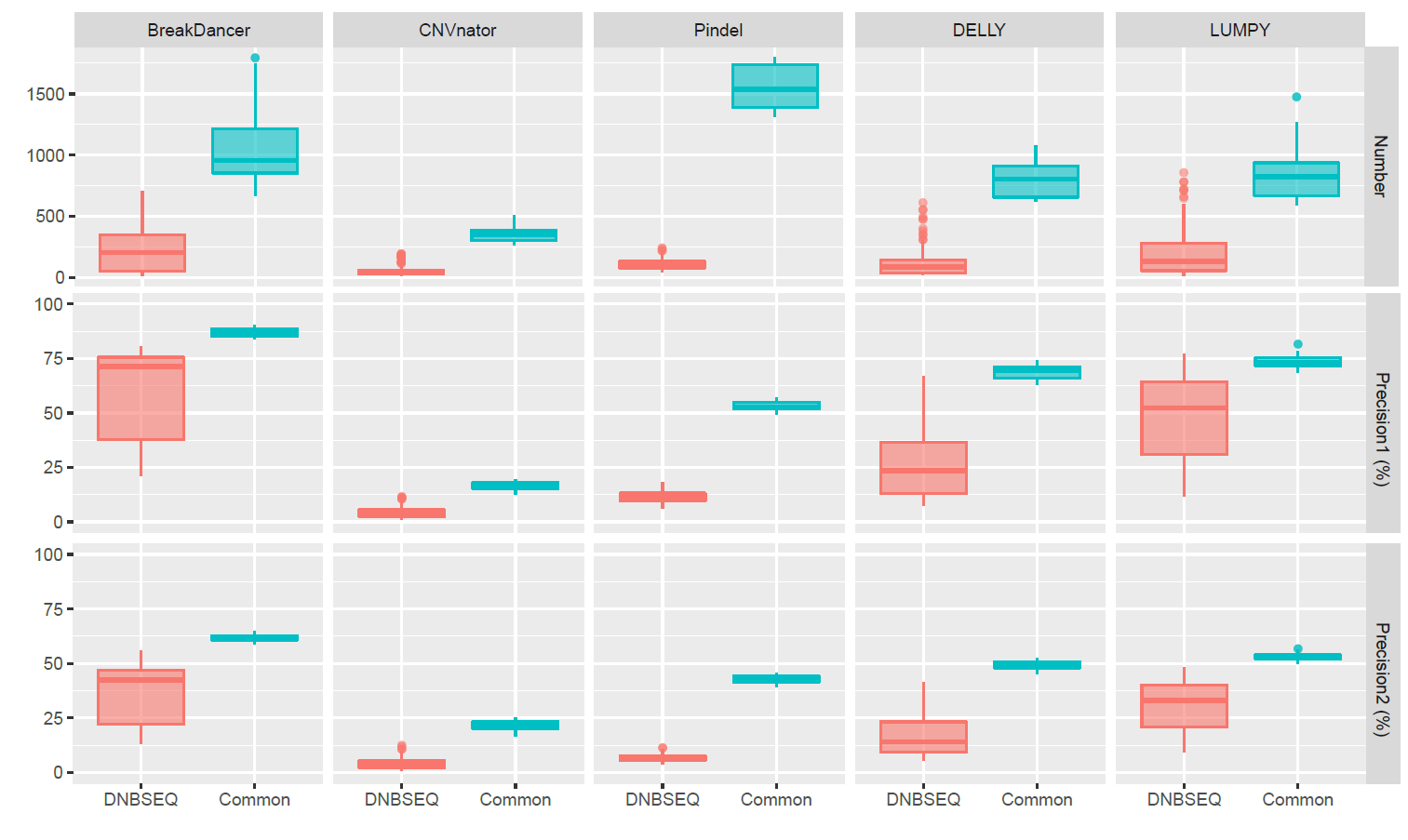


**Figure S5.** Comparison of CNVs by data on Illumina platforms. Box plot shows the number (upper), precision based on Benchmark1 (median) and precision based on Benchmark2 (lower) of common and specific CNVs between platforms by different tools (column). Precision1, precision based on Benchmark1; Precision2, precision based on Benchmark2.


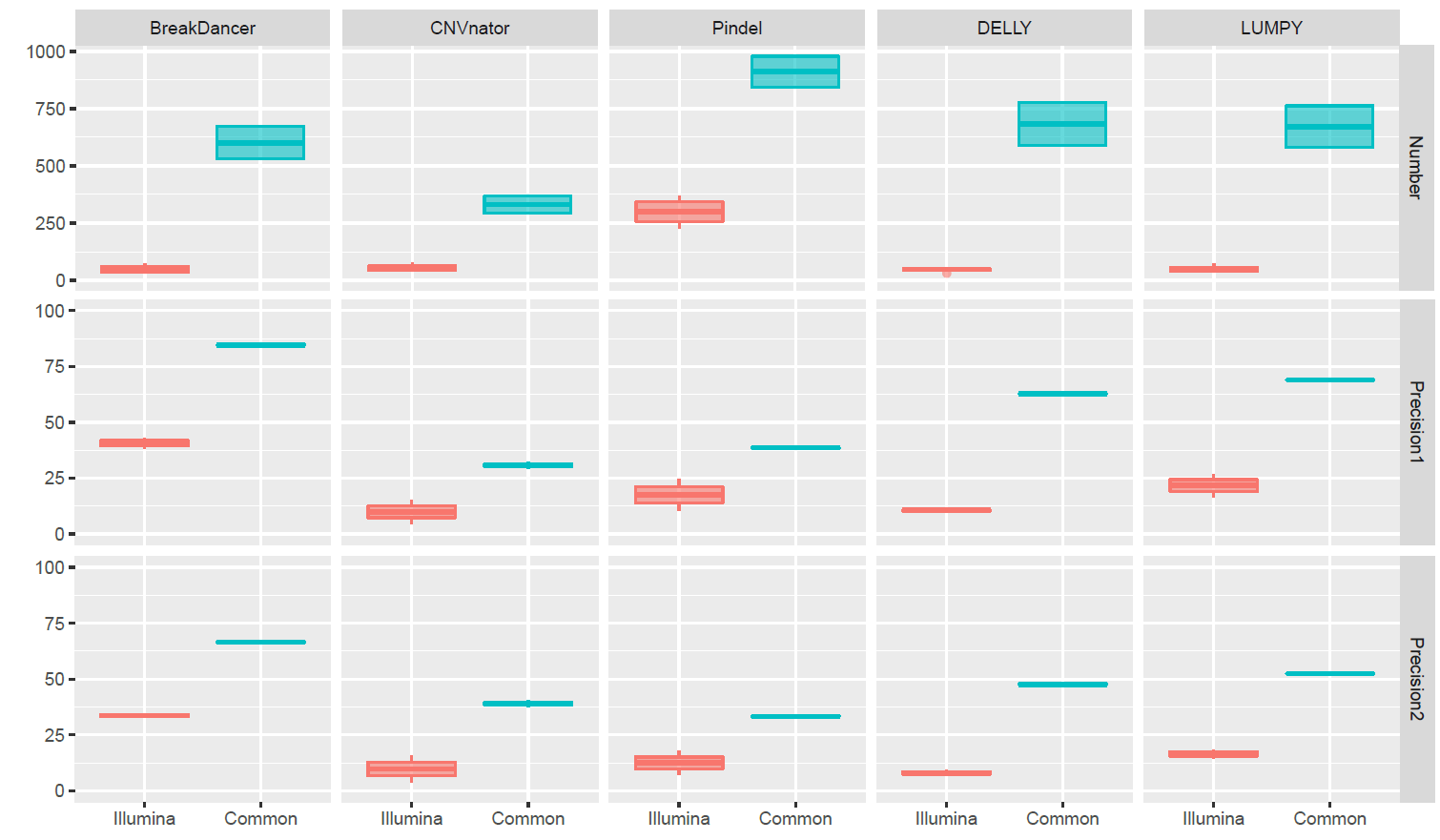


**Figure S6.** Comparison of the precision and sensitivity between two benchmarks on all 50 CNV sets. (A) Box plot shows the difference in precision between Benchmark1 (left) and Benchmark2 (right). (B) Box plot shows the difference in sensitivity between two benchmarks. Boxplot represents the rate of all 50 CNV sets, and dashed lines were drawn to connect the results based on the same dataset. **, P < 0.01; measured by the t-test. Benchmark1, data by Ryan et al., 2014; Benchmark2, data by Peter et al., 2015.


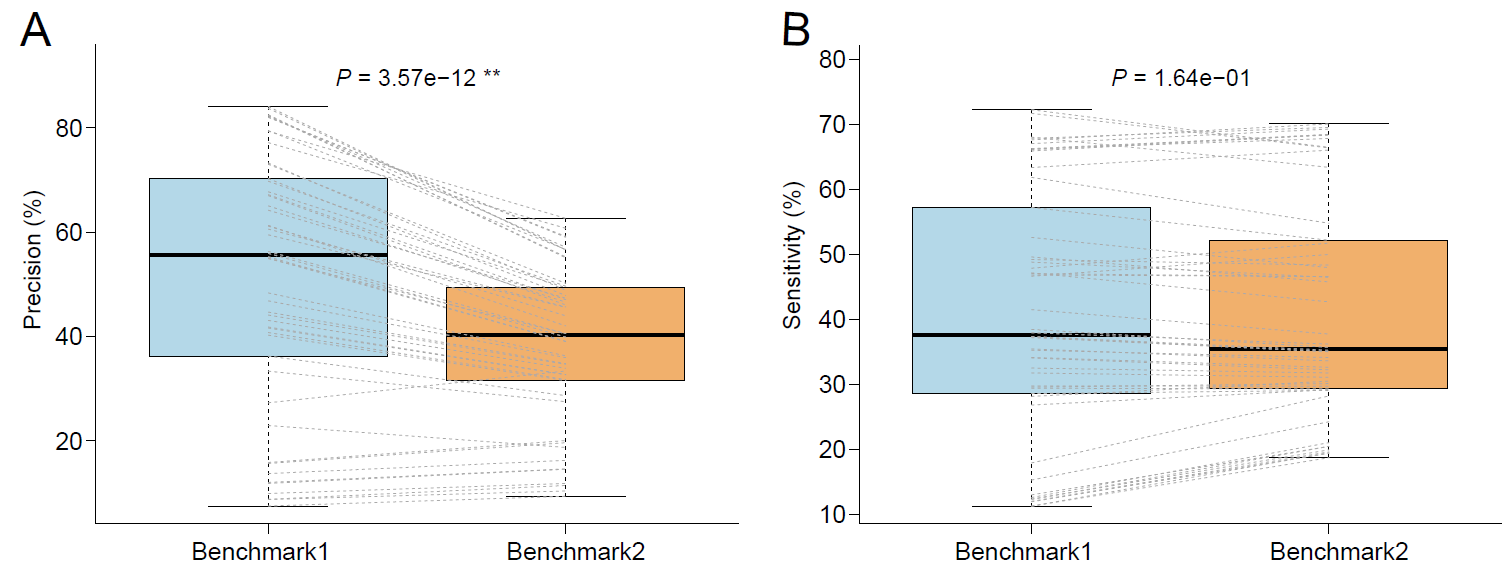


**Figure S7.** Novel, complete CNV benchmark of NA12878. (a) Histogram shows the number of CNVs with different lengths in the complete NA12878 CNV benchmark. (b) Pie shows the components of the complete NA12878 CNV benchmark. Labels without a plus sign, such as “Benchmark1”, “Benchmark2” and “Predicted”, represent a unique source of the CNV benchmark. Labels with a plus sign indicate that the CNV benchmark was provided by at least two sources (“Benchmark 1+Predicted” indicates that the CNV benchmark was provided by both Benchmark1 and Predicted benchmark). (c) Bar plot shows the precision, sensitivity and F1-score of DNBSEQ^TM^ platforms and Illumina platforms. Black lines show the standard error. Benchmark1, data by Ryan et al., 2014; Benchmark2, data by Peter et al., 2015; Predicted, a predicted CNV benchmark from CNVs in this study.


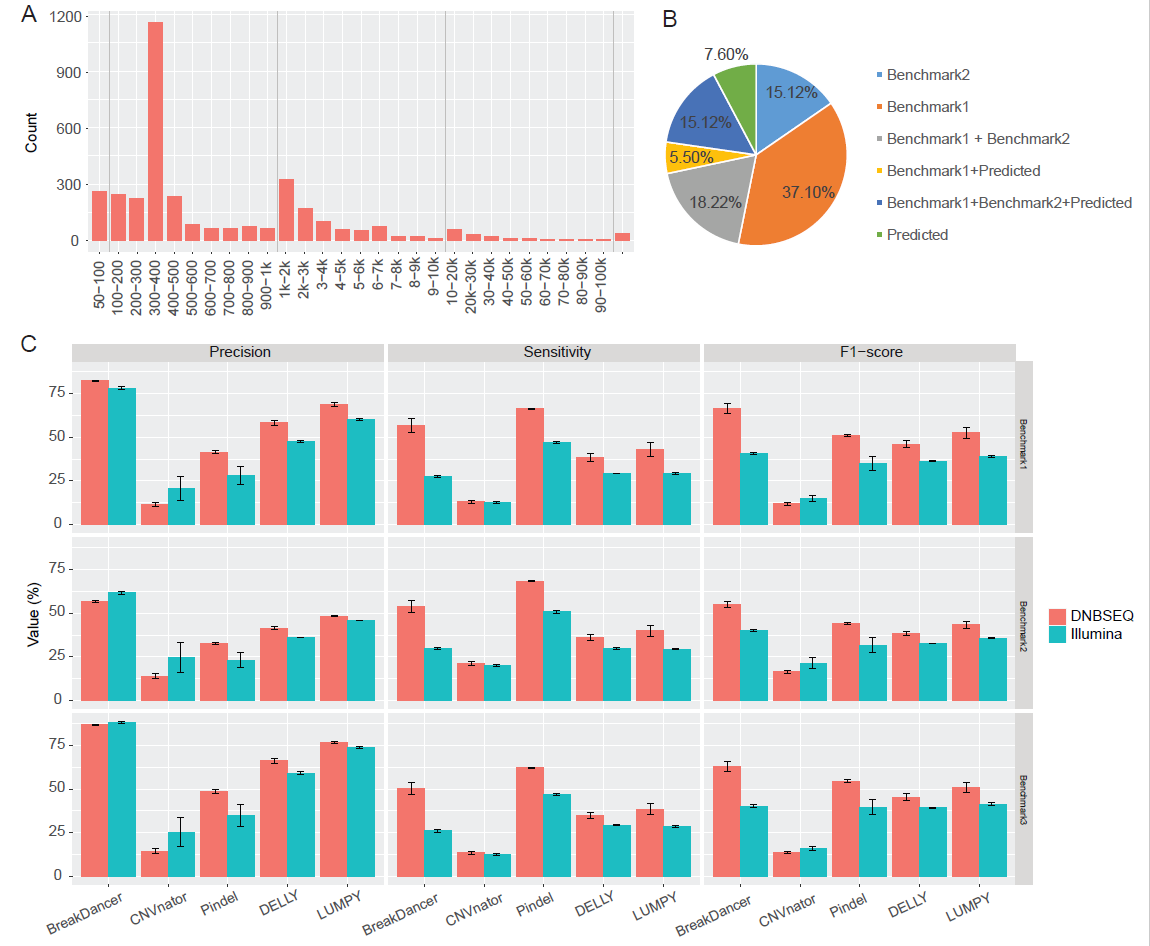


**Figure S8.** Comparison of two CNV benchmarks. Venn diagram shows the number and ratio of specific CNVs in each benchmark with a 90.00% threshold (A) or a 50.00% threshold (B). Benchmark1, data by Ryan et al., 2014; Benchmark2, data by Peter et al., 2015.


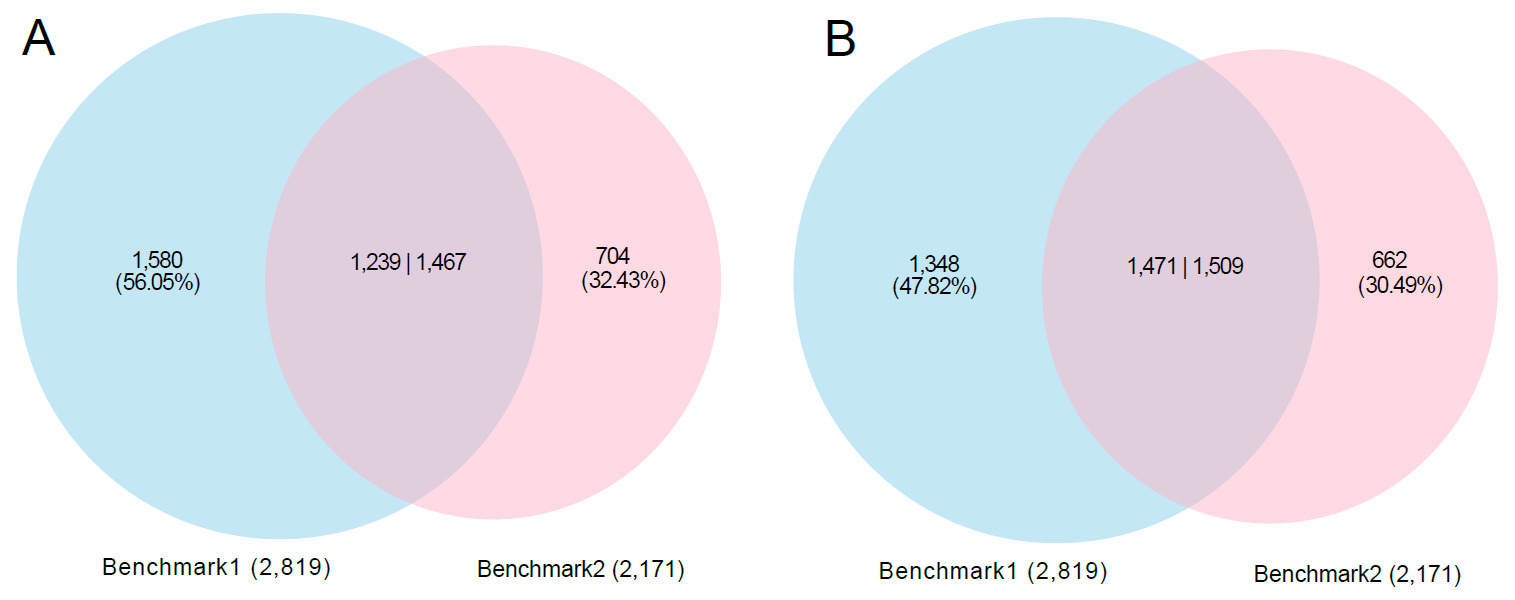


**Figure S9.** Summary of the distribution and precision of overlapping and non-overlapping CNVs. (a) Histogram shows the number (upper) and proportion (lower) of overlapping and non-overlapping CNVs (n=50) from ten datasets using five tools. (b, c) The comparison of precision between overlapping and non-overlapping CNVs with Benchmark1 (b) and Benchmark2 (c) is displayed below. Benchmark1, data by Ryan et al., 2014; Benchmark2, data by Peter et al., 2015.


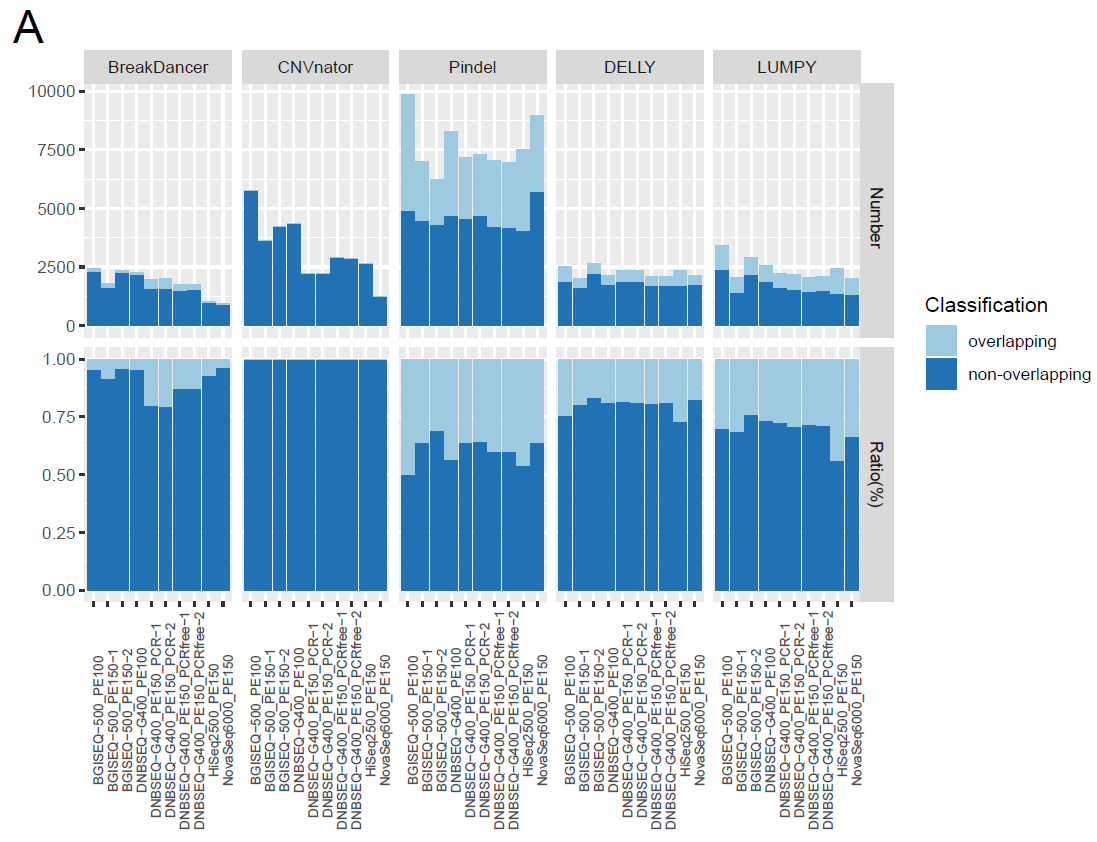


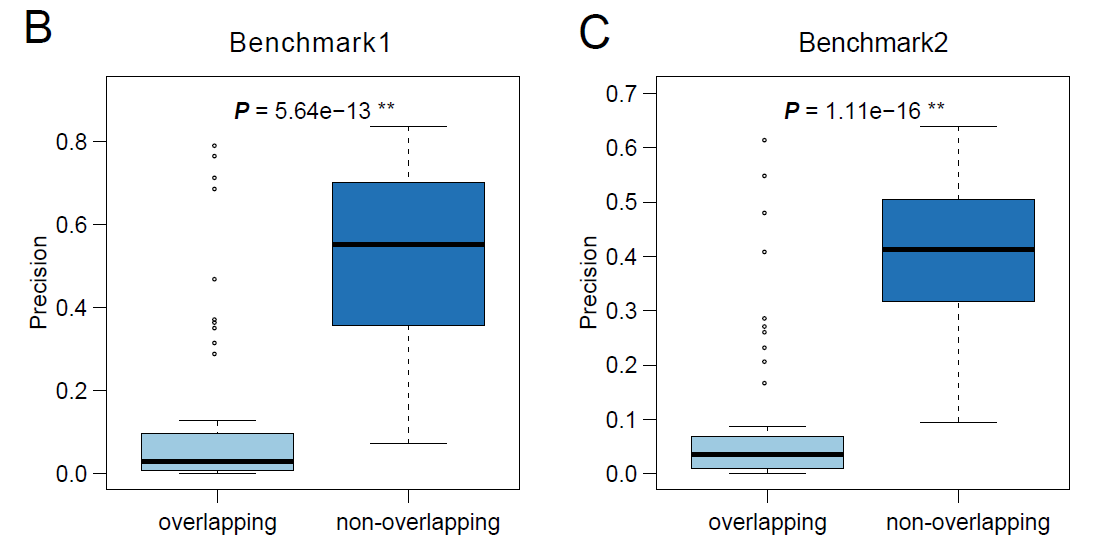

Supplement: Supplementary file 2 — Additional file 2. Figure S1: Consistency ratios of pairwise comparisons between all 50 CNV sets. Heatmap shows the consistency ratio distribution between any two CNV sets or benchmarks. Figure S2: Summary of the density distribution of CNV length for ten datasets using five tools. Each inner chart represents the CNV results of ten datasets detected by each tool. In each inner chart, the line plot shows the density (y-axis) of the CNV count at a certain CNV length (x-axis), and the two black vertical lines indicate the Alu elements (left) and the LINE1 elements (right). Figure S3: Annotation of CNVs across the genome. Histogram shows the number (upper) and proportion (lower) of CNVs occurring in different regions across the genome. CpG island: CGI. CpG island-shore: CGI-shore. Figure S4: Comparison of CNVs by data on DNBSEQTM platforms. Box plot shows the number (upper), precision based on Benchmark1 (median) and precision based on Benchmark2 (lower) of common and specific CNVs between platforms by different tools (column). Precision1, precision based on Benchmark1; Precision2, precision based on Benchmark2. Figure S5: Comparison of CNVs by data on Illumina platforms. Box plot shows the number (upper), precision based on Benchmark1 (median) and precision based on Benchmark2 (lower) of common and specific CNVs between platforms by different tools (column). Precision1, precision based on Benchmark1; Precision2, precision based on Benchmark2. Figure S6: Comparison of the precision and sensitivity between two benchmarks on all 50 CNV sets. (A) Box plot shows the difference in precision between Benchmark1 (left) and Benchmark2 (right). (B) Box plot shows the difference in sensitivity between two benchmarks. Boxplot represents the rate of all 50 CNV sets, and dashed lines were drawn to connect the results based on the same dataset. **, P < 0.01; measured by the t-test. Benchmark1, data by Ryan et al. 2014; Benchmark2, data by Peter et al. 2015. Figure S7: Novel, complete [file 12859_2020_3859_MOESM2_ESM.docx]
